# Erratum: Murrell, A., et al. Measuring Food Insecurity Using the Food Abundance Index: Implications for Economic, Health and Social Well-Being. *Int. J. Environ. Res. Public Health* 2020, *17*, 2434

**DOI:** 10.3390/ijerph17186495

**Published:** 2020-09-07

**Authors:** Audrey Murrell, Ray Jones

**Affiliations:** School of Business and David Berg Center for Ethics and Leadership, University of Pittsburgh, Pittsburgh, PA 15260, USA; rayjones@katz.pitt.edu

Since no human subjects were included in this research review [1], approval by the Institutional Review Board was not required. Consequently the authors would like to delete the following sentence:

Review and approval for conducting this research was submitted to the Institutional Review Board to ensure that all ethical standards for research were followed (Project Internal Case Number: 02907).
